# Domain Adaptation with Data Uncertainty Measure Based on Evidence Theory

**DOI:** 10.3390/e24070966

**Published:** 2022-07-13

**Authors:** Ying Lv, Bofeng Zhang, Guobing Zou, Xiaodong Yue, Zhikang Xu, Haiyan Li

**Affiliations:** 1 School of Computer Engineering and Science, Shanghai University, Shanghai 200444, China; lvying2016@shu.edu.cn (Y.L.); gbzou@shu.edu.cn (G.Z.); yswantfly@shu.edu.cn (X.Y.); xuzhikangnba@shu.edu.cn (Z.X.); 2 School of Computer and Information Engineering, Shanghai Polytechnic University, Shanghai 201209, China; 3 School of Computer Science and Technology, Kashi University, Kashi 844006, China; lihaiyan_2016@sjtu.edu.cn

**Keywords:** domain adaptation, transfer learning, evidence theory, uncertainty measure

## Abstract

Domain adaptation aims to learn a classifier for a target domain task by using related labeled data from the source domain. Because source domain data and target domain task may be mismatched, there is an uncertainty of source domain data with respect to the target domain task. Ignoring the uncertainty may lead to models with unreliable and suboptimal classification results for the target domain task. However, most previous works focus on reducing the gap in data distribution between the source and target domains. They do not consider the uncertainty of source domain data about the target domain task and cannot apply the uncertainty to learn an adaptive classifier. Aimed at this problem, we revisit the domain adaptation from source domain data uncertainty based on evidence theory and thereby devise an adaptive classifier with the uncertainty measure. Based on evidence theory, we first design an evidence net to estimate the uncertainty of source domain data about the target domain task. Second, we design a general loss function with the uncertainty measure for the adaptive classifier and extend the loss function to support vector machine. Finally, numerical experiments on simulation datasets and real-world applications are given to comprehensively demonstrate the effectiveness of the adaptive classifier with the uncertainty measure.

## 1. Introduction

In the field of machine learning research, supervised learning methods have already witnessed the outstanding performance in many applications. The key point of supervised learning is to collect sufficient labeled datasets for model training, which also limits the usage of supervised learning in scenarios with a lack of training data. Furthermore, data annotating is usually a time-consuming, labor-expensive, or even unrealistic task. To settle this situation, domain adaption (DA) is a promising methodology that aims to learn an adaptive classifier for the target domain tasks by making use of labeled data from source domains [1,2,3,4]. It has been applied in various fields successfully, such as object recognition [5,6], text classification [7,8], medical field [9,10], machine translation [11] and so on.

**Figure 1:**
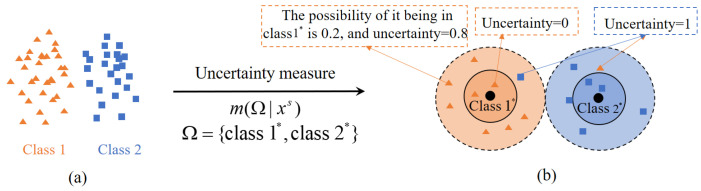
(**a**) Data distribution of source domain, (**b**) category distribution of source domain data in label space Ω of the target domain.

However, due to the mismatch between the source domain data and the target domain task, there is an uncertainty in DA when source domain data transfers to tasks of the target domain. As shown in Figure 1, in the target domain classification task, each source domain datum may no longer fully belong to a class in the label space of the target domain. The possibility of it being in class 1* is 0.2, and the uncertainty is 0.8, or the possibility of it being in class 1* is 0.9, and the uncertainty is 0.1. Unfortunately, the uncertainty of source domain data with respect to the target domain task is given less attention in DA. Ignoring uncertainty may result in an issue that the classifier does not fully match the target domain task, which weakens the model’s transfer performance.

Most DA research works adopted metric learning to minimize the data differences between the source and target domain for getting an adaptive classifier. Some works map the source and target data instances into a common feature space by minimizing the gap between the data distributions of the source and target domain, such as transfer component analysis (TCA) [12], correlation alignment (CORAL) [13], and scatter component analysis (SCA) [14]. Some works construct a loss function with the data differences as the constraint to train an adaptive classifier, such as joint adaptation network (JAN) [15], manifold embedded distribution alignment (MEDA) [16], and multi-representation adaptation network (MRAN) [17]. However, existing methods (1) cannot measure the uncertainty of source domain data about the target domain task, and (2) cannot accomplish effective training of adaptive classifiers with a data uncertainty measure.

The uncertainty is important for evaluating the adaptation degree of the source domain data about target tasks. The study of uncertainty has been successfully applied in traditional machine learning, such as bayesian-based uncertainty [18], evidence theory-based uncertainty [19], information entropy-based uncertainty [20], and granular computing-based uncertainty [21]. In particular, the evidence theory has been widely combined with machine learning methods to improve their ability to handle the uncertainty data [22,23,24,25,26].

To solve these problems, in this paper, we revisit the domain adaptation from source domain data uncertainty based on evidence theory and thereby devise a reliable adaptive classifier with the uncertainty measure. Specifically, we first construct an evidence net based on evidence theory for measuring the uncertainty of source domain data about the target domain classification task. It can calculate the proportion of uncertainty for each source domain instance in the target domain classification task. Second, we design a general loss function with the uncertainty measure for the adaptive classifier and extend the loss function to support vector machine (SVM). The contributions of this paper are summarized as follows.

- Designing an evidence net based on evidence theory to measure the uncertainty of source domain data about a target domain classification task.
- Designing a general loss function with uncertainty measure for learning of the adaptive classifier.
- Extending the SVM by the general loss function with uncertainty measure for enhancing its transferred performance.

The remainder of the paper is organized as follows. We start by reviewing related works in Section 2. Section 3 describes the evidence net that is built based on evidence theory for estimating the uncertainty. Section 4 extends the general loss function to SVM. Section 5 presents the experimental results to validate the efficiency of the proposed method. The conclusion about our exploratory work is also given in the last section.

## 2. Related Work

In this section, we discuss previous works on domain adaptation that minimizes the data difference between the source and target domain. In addition, we introduce the evidence theory that is most related to our work.

### 2.1. Domain Adaptation with Metric Learning

We will briefly introduce the domain adaptation with metric learning. These methods leverage the metric methods to reduce the data difference between two domains.

Maximum mean discrepancy (MMD) [27] takes advantage of the kernel trick, which can measure the data difference between the source domain and target domain. MMD is widely used in domain adaptation. Some state-of-the-art methods are proposed based on MMD. Pan and Yang et al. [12] propose the transfer component analysis (TCA) model based on MMD. The TCA utilizes the MMD to reduce the gap between the source domain and target domain. Long et al. [28] put forward the joint distribution adaptation (JDA) algorithm that uses the MMD to adapt both the marginal distribution and conditional distribution in domain adaptation. Muhammad Ghifary et al. [29] propose a neural network model that embeds the MMD regularization to reduce the distribution mismatch. Long et al. [30] propose a novel framework that is called adaptation regularization-based transfer learning (ARTL). The ARTL optimizes the structural risk functional, joint distribution adaptation of both the marginal, and conditional distributions by embedding the MMD regularization. Yan et al. [31] propose a weighted domain adaptation network (WDAN) by both incorporating the weighted MMD into CNN and taking into account the empirical loss on target samples.

Kullback–Leibler (KL) divergence [32] can measure data distribution differences between the source domain and target domain. Dai et al. [33,34] use the KL divergence to measure the difference between the source domain and target domain and uses the difference in co-clustering to improve the performance of transferring. Zhuang et al. [35] propose a supervised representation learning method based on a deep auto-encoder for domain adaptation. In the embedding layer, the authors use the KL divergence to keep the two distributions of source and target domains similar.

Jensen–Shannon (JS) divergence is similar to KL divergence and measures the difference between the source domain and target domain. However, the JS divergence solves the asymmetry problem of KL divergence. Joshua Giles et al. [36] use JS divergence to compare calibration trails with an electroencephalogram dataset for selecting the target user in domain adaptation. Subhadeep Dey et al. [37] employ JS divergence in Information Bottleneck clustering to find clusters in domain adaptation.

The Wasserstein distance derives from the optimal transport problem. It can be used to measure distances between two probability distributions. Shen et al. [38] reduce the discrepancy between the source domain and target domain by gradient property of the Wasserstein distance for improving transfer performance. Lee et al. [39] use the Wasserstein discrepancy between classifiers to align distributions in domain adaptation.

In summary, the core idea of most methods is to minimize the distribution difference between the source and target domain. However, they ignore the uncertainty between the source domain data and the target domain task.

### 2.2. Learning with Evidence Theory

Evidence theory can be considered a generalized probability [19,40]. It can represent and measure data uncertainty using mass function [41]. The evidence theory uses Dempster’s rule to finish uncertainty reasoning [42]. We will recall mass function and Dempster’s rule from evidence theory.

#### 2.2.1. Mass Function

Let $\Omega =\{z_{1},z_{2},\ldots ,z_{n}\}$ be a finite domain (set) that includes all possible answers to the decision problem, and the elements of the set are mutually exclusive and exhaustive. Ω is called the frame of discernment. In the classification problems, the element $z_{k}$ can be regarded as the *k*th category, and Ω can be considered as the sample space or label space. We denote the power-set as $2^{\Omega }$, and the cardinality of the power-set is $2^{\left|\Omega \right|}$.

The mass function m(·) is the Basic Belief Assignment (BBA) that represents the support degree of evidence, and m(·) is a mapping from $2^{\Omega }$ to the interval [0,1]. It satisfies the condition as follows

(1) $$\left\{\begin{array}{c} \sum_{A\in 2^{\Omega }}m\left(A\right)=1\\ m(\emptyset )=0 \end{array}\right.$$

where m(A) measures the support degree for proposition *A* itself and m(∅) represents that the empty set has no support degree. If m(A)>0, *A* is called a focal element. In classification problems, if $A=z_{k}$, m(A) can be interpreted as a support degree (possible) that instance belongs to class $z_{k}$. If A=Ω, m(A) can be interpreted as the total ignorance degree for classification results. In this paper, m(Ω) can be used to reflect the instance uncertainty.

For example, we assume a classification problem that distinguishes colors. The frame of discernment is Ω={red,green,blue}. The power-set is $2^{\Omega }=\{\emptyset ,\left\{red\right\},\left\{green\right\},\left\{bule\right\},$$\left\{red,green\right\},\left\{red,blue\right\}$, $\left\{green,blue\right\},\Omega \}$ and |Ω|=3, $2^{\left|\Omega \right|}=8$. m(green|x;E) represents the possibility that *x* belong to green based on evidence *E*. m(Ω|x;E) represents that we can not determine which class the sample belongs to. It reflects the instance uncertainty.

#### 2.2.2. Dempster’s Rule

Dempster’s rule reflects the combined effect of evidence. Let $m_{1}$ and $m_{2}$ be two mass functions induced by independent items of evidence. They can be combined using Dempster’s rule to form a new mass function defined as

(2) $$\left(m_{1}⊕m_{2}\right)\left(A\right)=\frac{1}{1-\kappa }\sum_{B\cap C=A}m_{1}\left(B\right)m_{2}\left(C\right),$$

for all A⊆Ω, A≠∅ and $\left(m_{1}⊕m_{2}\right)\left(\emptyset \right)=0$ (⊕ is the combination operator of Dempster’s rule). *k* is the degree of conflict between $m_{1}$ and $m_{2}$; it can be defined as

(3) $$\kappa =\sum_{B\cap C=\emptyset }m_{1}\left(B\right)m_{2}\left(C\right).$$

## 3. Uncertainty Measure in Domain Adaptation Based on Evidence Theory

In domain adaptation, the key problem of the uncertainty measure is how to evaluate the uncertainty in the target domain classification task for each source domain data. We consider that the lower uncertainty of instance represents less information loss in domain adaptation. To achieve this, we construct an evidence net based on evidence theory. It consists of two key steps (1) obtaining a trusty evidence set, and (2) designing the evidence net based on evidence theory. We describe them separately below.

### 3.1. Obtaining the Trusty Evidence Set

Let us consider a simple scenario with a large number of instances labeled source-domain $D^{s}$ and a small number of instances labeled target-domain $D_{l}^{t}$.

Given a source-domain instance $x^{s}$, its evidence set $\Phi^{t}$ consists of similar instances from the target domain and can be formulated as a neighborhood surrounding $x^{s}$.

(4) $$\Phi^{t}=\left\{x_{1}^{t},x_{2}^{t},\cdots ,x_{n}^{t}\right\},$$

in which $x_{1}^{t},x_{2}^{t},\cdots ,x_{n}^{t}$ are *n* target domain instances similar to the source domain instances $x^{s}$ and n>10. To ensure the validity of the evidence set, the discrepancy between a source-domain instance and the elements of its evidence set should be small. Motivated by this, we design the objective function of obtaining an evidence set for a source domain instance $x^{s}$ as

(5) $$\Phi^{t}=\underset{\Phi }{\arg\min}\;h\left(x^{s},\Phi \right),$$

in which the function h(·) measures the discrepancy between the $x^{s}$ of the source domain and the evidence set $\Phi^{t}$ in a reproducing kernel Hilbert Space (RKHS) H, h(·) is formulated as

(6) $$h\left(x^{s},\Phi^{t}\right)={∥\phi \left(x^{s}\right)-\frac{1}{|\Phi^{t}|}\sum_{x^{t}\in \Phi^{t}}\phi \left(x^{t}\right)∥}_{H}^{2},$$

where ϕ:X↦H is the feature mapping, and $|\Phi^{t}|$ is the number of elements in the evidence set. In this paper, we utilize the radial basis function kernel to construct the kernel Hilbert space,

(7) $$K\left(x^{t},x^{s}\right)=\phi {\left(x^{t}\right)}^{T}\phi \left(x^{s}\right)=\exp\left(-\gamma {∥x^{t}-x^{s}∥}^{2}\right),$$

in which ${∥x^{t}-x^{s}∥}^{2}$ is the Euclidean distance between two points and γ is a scaling parameter. Substituting $K\left(x^{t},x^{s}\right)$ into Equation (6), the function h(·) can be rewritten as

(8) $$h\left(x^{s},\Phi^{t}\right)=|\frac{1}{|\Phi^{t}{|}^{2}}\sum_{x_{1}^{t},x_{2}^{t}\in \Phi^{t}}K\left(x_{1}^{t},x_{2}^{t}\right)-\frac{2}{|\Phi^{t}|}\sum_{x^{t}\in \Phi^{t}}K\left(x^{s},x^{t}\right)|.$$

Based on the above analysis, the objective function of Equation (5) to obtain the evidence set can be specified as

(9) $$\Phi^{t}=\underset{\Phi }{\arg\min}|\frac{1}{{\left|\Phi \right|}^{2}}\sum_{x_{1}^{t},x_{2}^{t}\in \Phi^{t}}K\left(x_{1}^{t}x_{2}^{t}\right)-\frac{2}{\left|\Phi \right|}\sum_{x^{t}\in \Phi }K\left(x^{s},x^{t}\right)|.$$

The optimal evidence set $\Phi^{t}$ in Equation (9) can be solved by a greedy search on the labeled target domain.

### 3.2. Constructing Evidence Net Based on Evidence Theory

In the evidence theory, suppose that m(·|x;Φ) is the mass function, Ω is the label space, and Φ is the evidence set, the mass function m(Ω|x;Φ) can represent the uncertainty of *x* about the classification task. In domain adaptation, Ω comes from the label space of the target domain. In a built-up evidence set $\Phi^{t}$, from the target domain $D^{t}$, for instance, $x^{s}$, from source domain $D^{s}$, $m(\Omega |x^{s};\Phi^{t})$ can represent the uncertainty of the source domain instance $x^{s}$ about the target domain classification task.

In this section, motivated by evidential k-Nearest Neighbor [22] and neural network, we construct an evidence net based on Dempster’s rule to calculate $m(\Omega |x^{s};\Phi^{t})$. The details of the evidence net are described as follows.

According to Section 3.1, the evidence set $\Phi^{t}$ has been generated from the labeled target domain $D_{l}^{t}$. Given *k* classes, we decompose the evidence set $\Phi^{t}$ into different classes,

(10) $$\Phi^{t}=\left\{\Phi_{1}^{t},\Phi_{2}^{t},\ldots ,\Phi_{k}^{t}\right\},$$

where $\Phi_{k}^{t}=\left\{x_{k1}^{t},\ldots x_{kl}^{t}\right\}$ is the evidence subset in which all the target domain instances have the class label $z_{k}$, and $x_{kl}^{t}$ is the *l*th element in the evidence subset.

According to the decomposition of the evidence set $\Phi^{t}$ and Dempster’s rule, the evidence net can be represented in the connectionist formalism as a network with an input layer, three evidence layers $L_{1}$, $L_{2}$, and $L_{3}$, and an output layer.

**Figure 2:**
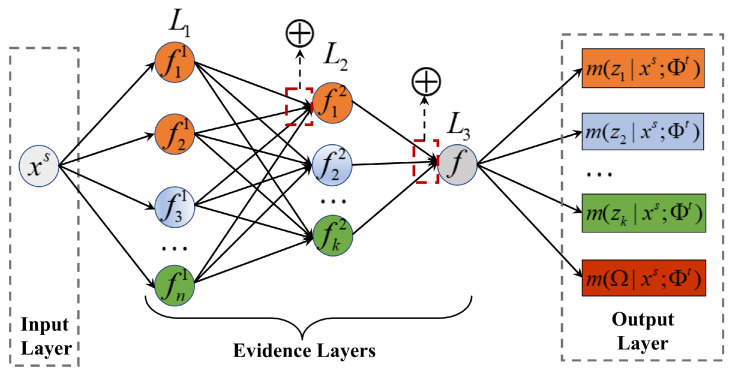
Evidence net architecture.

As shown in Figure 2, the input layer is an instance of source domain $x^{s}$, and the output layer is $m(z_{k}|x^{s};\Phi^{t})$ and $m(\Omega |x^{s};\Phi^{t})$. Each evidence layer $L_{i}\left(i=1,2,3\right)$ corresponds to one step of the procedure described as follows.

(1) Layer $L_{1}$ contains *n* nodes, and we denote the node of layer $L_{1}$ as $f_{i}^{1}\left(\cdot |x^{s};x_{i}^{t}\right)$. The input of the node is an instance $x^{s}$ from source domain $D^{s}$. At the fine-grained evidence level, given an element $x_{i}^{t}$ in an evidence subset, we compute $f_{i}^{1}\left(\cdot |x^{s};x_{i}^{t}\right)$ as

(11) $$f_{i}^{1}\left(\cdot |x^{s};x_{i}^{t}\right)=\left\{\begin{array}{c} m\left(z_{k}|x^{s};x_{i}^{t}\right)\\ m\left(\Omega |x^{s};x_{i}^{t}\right). \end{array}\right.$$

in which

(12) $$\begin{array}{cc} \; & m\left(z_{k}|x^{s};x_{i}^{t}\right)=\exp\left(-d\left(x^{s},x_{i}^{t}\right)\right),\\ \; & m\left(\Omega |x^{s};x_{i}^{t}\right)=1-\exp\left(-d\left(x^{s},x_{i}^{t}\right)\right), \end{array}$$

where d(·) is defined as follows

(13) $$d\left(x^{s},x_{i}^{t}\right)=K\left(x^{s},x^{s}\right)-2K\left(x^{s},x_{i}^{t}\right)+K\left(x^{t},x_{i}^{t}\right),$$

in which $K\left(\cdot \right)$ is the radial basis function kernel.

(2) Layer $L_{2}$ contains *k* nodes, and we denote the node as $f_{k}^{2}\left(\cdot |x^{s};\Phi_{k}^{t}\right)$. Using Dempster’s rule to combine $f_{i}^{1}\left(\cdot |x^{s};x_{i}^{t}\right)$ under single evidence $x^{t}\in \Phi_{k}^{t}$, we can obtain $f_{k}^{2}\left(\cdot |x^{s};\Phi_{k}^{t}\right)$ under the evidence subset $\Phi_{k}$.

(14) $$f_{k}^{2}\left(\cdot |x^{s};\Phi_{k}^{t}\right)=\underset{x^{t}\subseteq \Phi_{k}^{t}}{⨁}f^{1}\left(\cdot |x^{s};x^{t}\right)=\left\{\begin{array}{c} m\left(z_{k}|x^{s};\Phi_{k}^{t}\right)\\ m\left(\Omega |x^{s};\Phi_{k}^{t}\right), \end{array}\right.$$

in which

(15) $$\begin{array}{cc} \; & m\left(\Omega |x^{s};\Phi_{k}^{t}\right)=\underset{x^{t}\in \Phi_{k}^{t}}{⨁}m\left(\Omega |x^{s};x^{t}\right)=\prod_{x^{t}\in \Phi_{k}^{t}}m\left(\Omega |x^{s};x^{t}\right),\\ \; & m\left(z_{k}|x^{s};\Phi_{k}^{t}\right)=\underset{x^{t}\in \Phi_{k}^{t}}{⨁}m\left(z_{k}|x^{s};x^{t}\right)=1-\prod_{x^{t}\in \Phi_{k}^{t}}m\left(\Omega |x^{s};x^{t}\right). \end{array}$$

where the orthogonal sum ⨁ represents the combination operator of Dempster’s rule.

(3) In layer $L_{3}$, we denote the node as $f\left(\cdot |x^{s};\Phi^{t}\right)$. $f\left(\cdot |x^{s};\Phi^{t}\right)$ can be calculated under the entire evidence set $\Phi^{t}$ through accumulating $f_{j}^{2}\left(\cdot |x^{s};\Phi_{j}^{t}\right)$ under evidence subsets.

(16) $$f\left(\cdot |x^{s};\Phi^{t}\right)=\underset{\Phi_{k}\subseteq \Phi^{t}}{⨁}f^{2}\left(\cdot |x^{s};\Phi_{k}^{t}\right)=\left\{\begin{array}{c} m\left(z_{k}|x^{s};\Phi^{t}\right)\\ m\left(\Omega |x^{s};\Phi^{t}\right), \end{array}\right.$$

in which

(17) $$\begin{array}{cc} \; & m\left(z_{k}|x^{s};\Phi^{t}\right)=\underset{\Phi_{k}^{t}\subseteq \Phi^{t}}{⨁}m\left(z_{k}|x^{s};\Phi_{k}^{t}\right)=\frac{1}{\kappa }m\left(z_{k}|x^{s};\Phi_{k}^{t}\right)\prod_{j\neq k}m\left(\Omega |x^{s};\Phi_{j}^{t}\right),\\ \; & m\left(\Omega |x^{s};\Phi_{k}^{t}\right)=\underset{\Phi_{k}^{t}\subseteq \Phi }{⨁}m\left(\Omega |x^{s};\Phi_{k}^{t}\right)=\frac{1}{\kappa }\prod_{k=1}^{n}m\left(\Omega |x^{s};\Phi_{k}^{t}\right),\\ \; & \sum_{k\in \Omega }m\left(z_{k}|x^{s};\Phi^{t}\right)+m\left(\Omega |x^{s};\Phi^{t}\right)=1, \end{array}$$

where κ is a normalizing factor.

(18) $$\kappa =\sum_{k=1}^{n}m\left(z_{k}|x^{s};\Phi_{k}^{t}\right)\prod_{j\neq k}\;m\left(\Omega |x^{s};\Phi_{j}^{t}\right)\;+\;\prod_{k=1}^{n}m\left(\Omega |x^{s};\Phi_{k}^{t}\right).$$

$m(\Omega |x^{s};\Phi^{t})$ represents the proportion of uncertainty in the target domain classification task for the source domain instance $x^{s}$. $m(z_{k}|x^{s};\Phi^{t})$ represents the possibility that source domain instance $x^{s}$ belongs to class $z_{k}$ of the target domain. In this paper, we use $m(\Omega |x^{s};\Phi^{t})$ to measure the uncertainty of source domain data about the target domain task. Algorithm 1 summarizes the evidence net-based uncertainty measure of source domain data in domain adaptation.
**Algorithm 1** The uncertainty measure based on evidence net for source domain data**Input:** source domain $D^{s}$, labeled target domain $D_{l}^{t}$.
**Output:** source domain $D^{s}$ with uncertainty $m(\Omega |x^{s};\Phi^{t})$.

 1: **for all**
$x^{s}\in D^{s}$
**do**

 2: Generate an evidence set $\Phi^{t}$ for $x^{s}$ according to Equation (9).

 3: Estimate uncertainty $m(\Omega |x^{s};\Phi^{t})$ of $x^{s}$ based on the evidence net $f(\cdot |x^{s};\Phi^{t})$.

 4: **end for**

 5: **return**
$D^{s}$ with $m(\Omega |x^{s};\Phi^{t})$.


## 4. Learning Algorithm of Adaptive Classifier with Uncertainty Measure

Section 3 has successfully solved the uncertainty measure of source domain data for target domain tasks. In domain adaptation, another key issue is how to use the uncertainty to learn an adaptive classifier. To solve this problem, we propose a general loss function with an uncertainty measure.

The learning algorithm with uncertainty measure can be transformed into a problem of regularized risk minimization with uncertainty $R[m\left(\Omega |x^{s};\Phi^{t}\right),L\left(x^{s},z,w\right)]$. Thus, the general loss function of the learning algorithm with uncertainty can be written as

(19) $$R\left[m\left(\Omega |x^{s};\Phi^{t}\right),L\left(x^{s},z,w\right)\right]=\frac{1}{N}\sum_{i=1}^{N}\left(1-m\left(\Omega |x_{i}^{s};\Phi^{t}\right)\right)L\left(x_{i}^{s},z_{i},w\right)+\lambda ||w||,$$

where instance $x^{s}$ comes from source domain $D^{s}$, L(·) is loss function, and *w* is the parameter of the model. In order to verify its effectiveness, we extend the loss function with an uncertainty measure to support vector machine (SVM).

### 4.1. Support Vector Machine with Uncertainty Measure (SVMU)

Based on the general loss function, we propose an improved support vector machine with an uncertainty measure (SVMU), which integrates the uncertainty of the source domain instance about the target domain task to SVM. The SVM uses only one penalty factor to control the balance between margin maximization and misclassification. However, in domain adaptation, due to domain differences, the classification hyperplane controlled by only one penalty factor cannot effectively distinguish classes of the target domain. The SVMU can change the penalty factor by the uncertainty measure. It makes the instances of the source domain that are beneficial to the target domain classification task become the new support vectors and diminishes the importance of some instances that have negative effects. Thus, SVMU is more flexible and superior in domain adaptation than SVM. The details of SVMU are described as follows.

SVM maps the input points into a high-dimensional feature space and finds a separating hyperplane that maximizes the margin between two classes in this space. According to the general loss function, Equation (19), the optimization problem for SVMU is then regarded as the solution to

(20) $$\min_{w,b,\xi }\frac{1}{2}{∥w∥}^{2}+C\sum_{i=1}^{N}\left(1-m\left(\Omega |x_{i}^{s};\Phi^{t}\right)\right)\xi_{i},$$

subject to

(21) $$\begin{array}{cc} \; & z_{i}\left(w\cdot \phi (x_{i}^{s})+b\right)\geq 1-\xi_{i}\;i=1,2,\cdots ,N,\\ \; & \xi_{i}\geq 0\;i=1,2,\cdots ,N, \end{array}$$

where parameter $\xi_{i}$ is the slack variable. C>0 is the penalty factor, which controls the trade-off between the slack variable penalty and the margin. ϕ(·) denotes a fixed feature-space transformation. *b* is the bias parameter.

To solve this optimization problem, we construct the Lagrangian function

(22) $$\begin{array}{cc} \; & L\left(w,b,\xi ,\sigma ,\lambda \right)=\frac{1}{2}{∥w∥}^{2}+C\sum_{i=1}^{N}\left(1-m\left(\Omega |x_{i}^{s};\Phi^{t}\right)\right)\xi_{i}\\ \; & -\sum_{i=1}^{N}\sigma_{i}\left(z_{i}\left(w\cdot \phi \left(x_{i}^{s}\right)+b\right)-1+\xi_{i}\right)-\sum_{i=1}^{N}\lambda_{i}\xi_{i}, \end{array}$$

To find the saddle point of L(w,b,ξ,σ,λ), the parameters satisfy the following conditions

(23) $$\begin{array}{cc} \; & \frac{\partial L(w,b,\xi ,\sigma ,\lambda )}{\partial \xi_{i}}=\left(1-m\left(\Omega |x_{i}^{s};\Phi^{t}\right)\right)*C-\sigma_{i}-\lambda_{i}=0,\\ \; & \frac{\partial L(w,b,\xi ,\sigma ,\lambda )}{\partial w}=w-\sum_{i=1}^{N}\sigma_{i}z_{i}\phi \left(x_{i}^{s}\right)=0,\\ \; & \frac{\partial L(w,b,\xi ,\sigma ,\lambda )}{\partial b}=-\sum_{i=1}^{N}\sigma_{i}z_{i}=0. \end{array}$$

By applying these conditions to the Lagrangian function (22), problem (20) can be transformed into

(24) $$\min_{w,b,\xi }L\left(w,b,\xi ,\sigma ,\lambda \right)=-\frac{1}{2}\sum_{i=1}^{N}\sum_{j=1}^{N}\sigma_{i}\sigma_{j}z_{i}z_{j}K\left(x_{i}^{s},x_{j}^{s}\right)+\sum_{i=1}^{N}\sigma_{i},$$

subject to

(25) $$\sum_{i=1}^{N}\sigma_{i}z_{i}=0,\;0\leq \sigma_{i}\leq \left(1-m\left(\Omega |x_{i}^{s};\Phi^{t}\right)\right)*C,$$

where K(·) is a kernel function

(26) $$K\left(x_{i}^{s},x_{j}^{s}\right)=\phi {\left(x_{i}^{s}\right)}^{T}\cdot \phi \left(x_{j}^{s}\right),$$

and the KKT conditions are defined as

(27) $$\begin{array}{cc} \; & \sigma_{i}^{*}\left(z_{i}\left(w^{*}\cdot \phi \left(x_{i}^{s}\right)+b^{*}\right)-1+\xi_{i}^{*}\right)=0,\\ \; & \left(\left(1-m\left(\Omega |x_{i}^{s};\Phi^{t}\right)\right)*C-\sigma_{i}^{*}\right)\xi_{i}^{*}=0, \end{array}$$

The optimal solution of (24) can be denoted as $\sigma^{*}=\left(\sigma_{1}^{*},\sigma_{2}^{*},\cdot ,\sigma_{N}^{*}\right)$, where $x_{i}^{s}$ corresponding to $\sigma_{i}^{*}>0$ is a support vector. The support vector $x_{i}^{s}$ falls exactly on the margin boundary if $0<\sigma_{i}^{*}<\left(1-m\left(\Omega |x_{i}^{s};\Phi^{t}\right)\right)*C$. If $\sigma_{i}^{*}=\left(1-m\left(\Omega |x_{i}^{s};\Phi^{t}\right)\right)*C$, $0<\xi_{i}<1$, then the classification is correct, and $x_{i}^{s}$ is between the boundary and the hyperplane. If $\alpha_{i}^{*}=\left(1-m\left(\Omega |x_{i}^{s};\Phi^{t}\right)\right)*C$ and $\xi_{i}=1$, then $x_{i}^{s}$ is on the classification hyperplane; if $\alpha_{i}^{*}=\left(1-m\left(\Omega |x_{i}^{s};\Phi^{t}\right)\right)*C$ and $\xi_{i}>1$, then $x_{i}^{s}$ is on the misclassified side of the classification hyperplane.

In the traditional SVM, the only penalty factor *C* controls the balance between margin maximization and misclassification. A larger *C* allows the SVM to have fewer misclassification and a narrower margin. Conversely, a smaller *C* makes the SVM ignore more training points and obtains a larger margin. Due to the existing uncertainty of the source domain data about the target domain task, with only one penalty factor, it is difficult to control the balance between margin maximization and misclassification in the target domain task. This may result in negative transfer when using SVM as the classifier.

**Figure 3:**
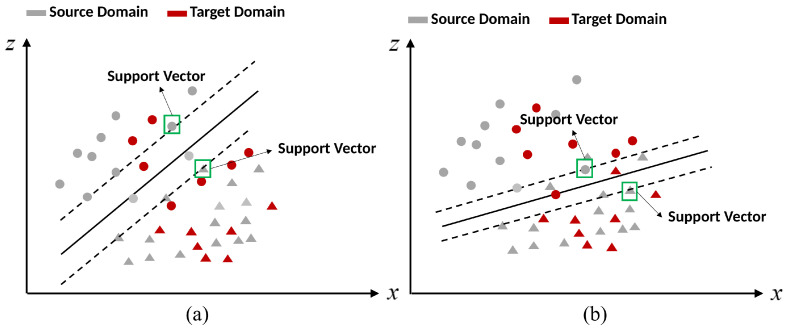
Schematic diagram: (**a**) Classification hyperplane is generated by SVM. (**b**) Classification hyperplane is generated by SVM with uncertainty.

Based on the above analysis, applying uncertainty to SVM, it can be found that the single penalty factor *C* becomes $(1-m\left(\Omega |x_{i}^{s};\Phi^{t}\right))*C$, whose number of penalty factors increases from one to the number of source domain instances. Each support vector corresponds to a penalty factor $(1-m\left(\Omega |x_{i}^{s};\Phi^{t}\right))*C$ with an uncertainty measure instead of corresponding to a single constant value *C*. Thus, the selection of support vectors does not rely on a single penalty factor but is determined by the uncertainty $m(\Omega |x_{i}^{s};\Phi^{t})$ of each source domain instance with respect to the target domain task. As shown in Figure 3, changing the penalty factor *C* by uncertainty $m(\Omega |x_{i}^{s};\Phi^{t})$ can make the instances of the source domain that are beneficial to the target domain classification task become the new support vectors and diminish the importance of some instances that have negative effects. The classification hyperplane that is generated by these new support vectors is suited to discriminate the target data. Thus, integrating the uncertainty to SVM can adjust the classification hyperplane to suit the target domain task.

## 5. Experiments

In the experiments, we evaluate the adaptive classifier with an uncertainty measure on various kinds of data, including texts and images. The descriptions of the datasets are listed below.

Amazon product reviews dataset [43] is the benchmark text corpora widely used for domain adaptation evaluation. The reviews are about four product domains: books (denoted as *B*), dvds (denoted as *D*), electronics (denoted as *E*), and kitchen appliances (denoted as *K*). Each review is assigned to a sentiment label, −1 (negative review) or +1 (positive review), based on the rating score given by the review author. In each domain, there are 1000 positive reviews and 1000 negative reviews. In this dataset, we construct 12 cross-domain sentiment classification tasks: B→D, B→E, B→K, D→B, D→E, D→K, E→B, E→D, E→K, K→B, K→D, K→E, where the word before an arrow corresponds to the source domain and the word after an arrow corresponds with the target domain. In each cross-domain classification task, we extract the features of the texts by using the word2vec tool.

Office+Caltech dataset [44] is commonly used for the task of visual object recognition in domain adaptation. It includes four domains: Amazon (denoted as *A*, images downloaded from online merchants), Webcam (denoted as *W*, low-resolution images from a web camera), DSLR (denoted as *D*, high-resolution images from a digital SLR camera), and Caltech-256 (denoted as *C*). The dataset includes 10 classes: backpack, touring bike, calculator, head Caltech, phones, computer keyboard, laptop-101, computer monitor, computer mouse, coffee mug, and video projector. There are 8 to 151 samples per category per domain and 2533 images in total. In this dataset, we construct 12 cross-domain multi-classification tasks: A→C, A→D, A→W, C→A, C→D, C→W, D→A, D→C, D→W, W→A, W→C, and W→D.

In the experiment, for each domain adaptation classification task, we use the classification accuracy of the target domain as the evaluation criterion. Suppose $D^{t}$ is the target domain dataset,

(28) $$\;\mathrm{Accuracy}\;=\frac{\left|\{x:x\in D^{t}\wedge v\left(x\right)=y\}\right|}{\left|\{x:x\in X\}\right|},$$

where *y* is the ground truth label of *x*, and v(x) is the label predicted by the classifier.

### 5.1. Comparative Studies

To evaluate the transfer performance of SVM with the uncertainty measure (SVMU), we compared it with 9 domain adaptation methods on Amazon product reviews dataset and Office+Caltech datasets, respectively. The methods of comparison include transfer component analysis (TCA) [12], correlation alignment (CORAL) [13], geodesic flow kernel (GFK) [44], joint distribution adaptation (JDA) [28], kernel mean matching (KMM) [45], metric transfer learning (MTLF) [46], scatter component analysis (SCA) [14], practically easy transfer learning (EasyTL) [47], and Wasserstein distance-guided representation learning (WDGAL) [38].

(1) Testing on Amazon product reviews dataset

In this testing, we evaluate SVM with an uncertainty measure (SVMU) on the Amazon product reviews dataset. The classification accuracies of the comparative study are listed in Table 1.

As shown in Table 1, the average classification accuracy of SVMU on the 12 tasks is 82.80%. The performance improvement is 6.17%, 12.77%, 9.28%, 6.04%, 5.19%, 12.47%, 2.70%, 3.33%, and 0.9% compared to baseline method. The average classification accuracy of TCA, CORAL, GFK, JDA, and KMM are 76.63%, 70.03%, 73.52%, 76.76%, and 77.61% on Amazon product reviews. These methods aim to minimize the different between the source and target domains, while ignoring the uncertainty of the instances in the source domain with respect to the task. Although they can find a representation space with the greatest commonality between the source and target domains, they cannot determine whether the source domain instance is suitable for the target domain task. This limits the performance of these methods. The performance improvement of our method is 6.17%, 12.77%, 9.28%, 6.04%, and 5.19% compared to them. In results of text classification, since these results are obtained from a larger number of datasets, it can convincingly verify that SVMU is reliable and effective for classifying cross-domain text accurately.

(2) Testing on Office+Caltech datasets

In this testing, we evaluate SVM with an uncertainty measure (SVMU) on Office+Caltech datasets. In each cross-domain classification task, we extract the features of images by speeded up robust features (SURF). The classification accuracies of the comparative study are listed in Table 2.

It is obvious that SVMU achieves better performance than the methods of comparison on Office+Caltech datasets. Specifically, the average classification accuracy of SVMU on 12 cross-domain classification tasks is 50.60%, which gains significant performance improvements of 4.16%, 4.1%, 5.22%, 4.04%, 4.55%, 4.63%, 3.23%, and 2.93% compared to the baseline methods. The experimental results reveal that the improved SVM with the uncertainty measure is reliable and effective in cross-domain image classification tasks.

### 5.2. Effectiveness Verification of Uncertainty Measure

In this experiment, we verify the effectiveness of the uncertainty measure from three views: (1) Testing on synthetic data, visualizing the classification hyperplane of an adaptive classifier with and without an uncertainty measure. (2) Testing on real-world datasets, comparing the performance of SVM with and without an uncertainty measure. (3) Case study, explaining the role of uncertainty measure.

#### 5.2.1. Testing on Synthetic Data

In order to demonstrate the effectiveness of the adaptive classifier with an uncertainty measure in domain adaptation, we visualize the classification hyperplane of an adaptive classifier on a synthetic dataset. The synthetic dataset is generated from a Gaussian distribution $x∼N\left(\mu ,\sigma \right)$, where μ and σ are the mean and standard deviation, respectively. We apply different μ and σ to generate the data from the source domain and target domain. In the dataset, the source domain and target domain consist of two-dimensional data points under two classes, and each class has 500 data points. The source domain is marked by a pentagram, and the target domain is marked by a triangle. Class 1 is marked in orange, and Class 2 is marked in dark slate-gray.

**Figure 4:**
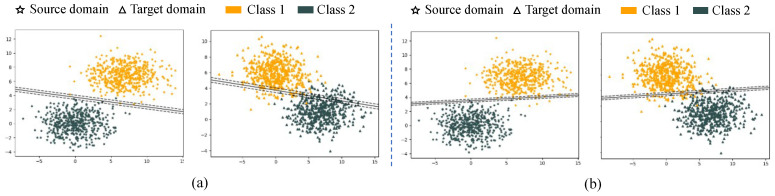
Results on synthetic data: (**a**) Classification hyperplane is generated by SVM. (**b**) Classification hyperplane is generated by SVM with uncertainty.

In Figure 4, (a) and (b) show the classification hyperplanes that are generated based on the source domain by SVM and SVMU, respectively. Due to the difference in data distribution between the source and target domains, the classification hyperplane generated by SVM cannot accurately distinguish the categories of the target domain and cannot satisfy the domain adaptation task. In contrast, the classification hyperplane generated by SVMU can accurately classify the target domain categories, and the classification results are shown in (a) and (b). The experimental results are consistent with the conclusions about SVMU in Section 4.1. Therefore, the uncertainty measure is effective and can improve the transfer performance of the adaptive classifier.

#### 5.2.2. Testing on Real-World Datasets

To further explain the effectiveness of the adaptive classifier with uncertainty, we compare the SVM with and without uncertainty on the Amazon product reviews dataset.

**Figure 5:**
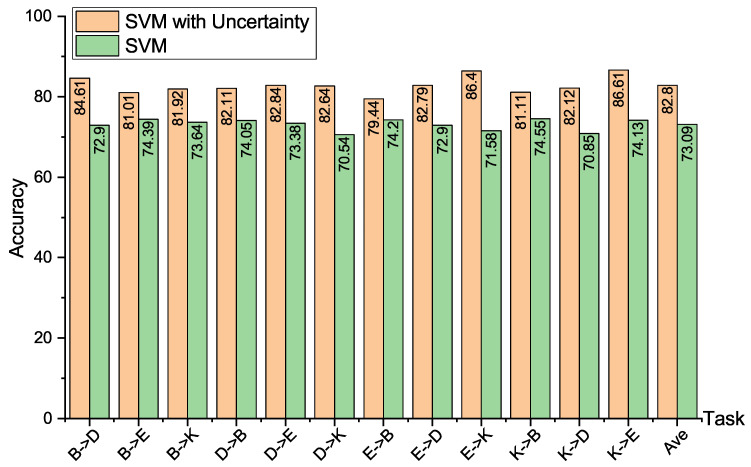
Cross-domain sentiment classification accuracies on Amazon product reviews generated by SVM with and without uncertainty.

Figure 5 shows the result of SVM with and without uncertainty on the Amazon product reviews dataset; it is obvious that in all the cross-domain text tasks, SVMU achieves better performance than SVM. SVMU improves the transfer accuracy over SVM on the 12 subtasks by 11.71%, 6.62%, 8.28%, 8.06%, 9.46%, 12.1%, 5.24%, 9.89%, 14.82%, 6.56%, 11.27%, and 12.48%. Comparing the average classification accuracy, SVMU improves the average classification accuracy by 9.71% over SVM. The above results show that it is effective at enhancing the transfer performance of the adaptive classifier by introducing the uncertainty between the source domain data and target domain task.

#### 5.2.3. Case Study

Based on the above sub-experiments, it can be verified that the uncertainty measure is able to enhance adaptive classifier transfer performance. To explain the role of the uncertainty measure on the transfer process for an adaptive classifier, we use the Caltech-256 image data (complex background) as the source domain and the Amazon image data (no background) as the target domain. When the Caltech-256 dataset transfers to the Amazon dataset, the uncertainty values of some instances in the backpack and bicycle categories in the Caltech-256 dataset are shown.

**Figure 6:**
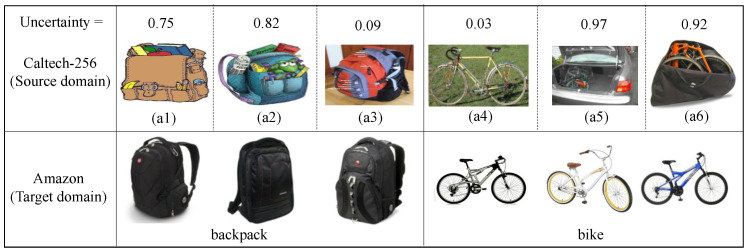
The uncertainty of the category ’backpack’ and ’bike’ in source domain *C* about the target domain *A* classification task.

As shown in Figure 6, for images (a1) to (a6) in the Caltech-256 dataset, it can be found that (a1) and (a2) are cartoon images of a backpack, and (a5) and (a6) are bicycles with obscure features. These instances are not significantly helpful for the target domain classification task. On the contrary, in (a3) and (a4), the features of the backpack and bicycle are obvious and beneficial for the target domain classification task.

We use the evidence net to calculate the uncertainty between (a1)–(a6) and the target domain task; as shown in Figure 6, we can find that the uncertainties of (a1), (a2), (a5), and (a6) calculated by the evidence network are high; 0.75, 0.82, 0.97, and 0.92, respectively. The uncertainties of (a3) and (a4) are low, at 0.09 and 0.03, respectively. When the Caltech-256 dataset transfers to the Amazon dataset, the images (a1)–(a6) no longer fully belong to the category of backpack and bicycle. (a1), (a2), and (a3) belong to the backpack category with the possibilities 0.25, 0.18, and 0.91, and 0.75, 0.82, and 0.09 are the uncertainties. (a4), (a5), and (a6) belong to the bicycle category with the possibilities 0.97, 0.03, and 0.08, and 0.03, 0.97, and 0.92 are the uncertainties. Based on the above results, it can be found that our proposed uncertainty measure is consistent with people’s cognition. Therefore, the uncertainty can accurately measure the adaptability of instances with respect to the target domain task.

## 6. Conclusions

In this article, based on evidence theory, we revisited the domain adaptation from source domain data uncertainty and thereby devised a reliable adaptive classifier with the uncertainty measure. Specifically, for solving the uncertainty measure between the source domain data and target domain tasks, we designed an evidence net based on evidence theory. To solve the problem of model learning with a data uncertainty measure, we proposed a general loss function with an uncertainty measure for an adaptive classifier and extended the loss function to support vector machine. Experiments on the text dataset and image dataset validate that the proposed uncertainty measure is effective at improving the transfer performance of an adaptive classifier. In the future, we plan to extend the classifier with the uncertainty measure to handle the domain adaptation with multiple source domains and the domain adaptation on open sets.

## Figures and Tables

**Table 1:** Cross-domain sentiment classification accuracies of Amazon product reviews generated by SVMU and baseline methods.

Task	SVMU	TCA	CORAL	GFK	JDA	KMM	MTLF	SCA	EasyTL	WDGAL
B→D	**84.61**	77.76	70.76	75.76	77.26	83.76	68.59	81.56	79.80	83.05
B→E	**81.01**	75.54	66.21	72.00	75.93	79.02	69.63	78.08	79.70	80.09
B→K	81.92	78.74	70.00	73.50	78.09	75.90	72.74	79.09	80.90	**85.45**
D→B	82.11	76.05	73.05	71.85	77.65	80.50	70.70	**82.35**	79.90	80.72
D→E	**82.84**	76.38	68.70	68.96	76.03	68.51	71.90	78.82	80.80	82.26
D→K	82.64	79.34	71.96	75.70	78.29	76.45	74.18	80.39	82.00	**85.23**
E→B	**79.44**	73.35	69.90	72.60	72.65	73.70	69.20	77.00	75.00	77.22
E→D	**82.79**	73.66	65.71	71.11	72.16	77.86	70.73	77.26	75.30	78.28
E→K	86.40	79.74	72.35	76.20	80.14	80.39	71.36	84.63	84.90	**88.16**
K→B	**81.11**	73.05	67.45	73.75	75.05	74.25	66.04	78.90	76.50	77.16
K→D	**82.12**	77.26	68.61	74.21	77.56	75.96	70.31	77.46	76.30	78.89
K→E	**86.61**	78.74	75.68	76.58	80.32	85.00	68.58	85.65	82.50	86.29
Average	**82.80**	76.63	70.03	73.52	76.76	77.61	70.33	80.10	79.47	81.90

**Table 2:** Cross-domain classification accuracies on Office+Caltech image datasets (SURF features) generated by SVMU and baseline methods.

Task	SVMU	TCA	CORAL	GFK	JDA	KMM	MTLF	SCA	EasyTL
A→C	**51.55**	47.76	45.37	40.25	49.36	45.41	45.37	48.29	43.01
A→D	44.31	41.12	43.75	43.31	42.49	41.40	41.38	44.21	**45.85**
A→W	**47.28**	44.63	44.78	43.98	45.97	42.85	42.59	43.90	40.68
C→A	**63.29**	58.20	53.59	51.20	54.78	50.10	54.17	53.74	50.10
C→D	44.00	41.40	46.22	42.85	43.22	43.58	40.69	39.49	**48.41**
C→W	**46.44**	42.64	43.73	40.68	41.69	43.81	46.10	43.56	42.49
D→A	**63.29**	52.15	58.81	52.05	53.09	58.60	59.92	57.72	61.94
D→C	**51.33**	49.70	48.01	48.28	45.52	47.81	45.73	50.32	51.17
D→W	**46.44**	46.10	44.40	45.59	43.49	44.45	43.50	42.81	44.49
W→A	**63.29**	58.06	56.20	59.75	56.78	52.15	51.07	60.48	60.18
W→C	**51.22**	45.30	42.08	48.72	49.17	49.81	49.38	50.63	49.65
W→D	**47.77**	43.26	44.08	40.89	46.17	45.62	44.76	46.36	47.07
Average	**51.68**	47.52	47.58	46.46	47.64	47.13	47.05	48.45	48.75

## Data Availability

Not applicable.
